# Australian Podiatry Research in Gerontology: A Bibliometric Analysis

**DOI:** 10.1002/jfa2.70158

**Published:** 2026-04-29

**Authors:** Hylton B. Menz, Ameer Nor Azhar, Shan M. Bergin, Peta E. Tehan, Matthew R. Carroll

**Affiliations:** ^1^ Discipline of Podiatry School of Allied Health Human Services and Sport La Trobe University Melbourne Victoria Australia; ^2^ Department of Surgery School of Clinical Sciences Faculty of Medicine Nursing and Health Sciences Monash University Clayton Victoria Australia; ^3^ School of Allied Health, Faculty of Health and Environmental Sciences Auckland University of Technology Auckland New Zealand

**Keywords:** bibliometrics, geriatrics, podiatry

## Abstract

**Background:**

To conduct a bibliographic analysis of English language research pertaining to gerontology by Australian resea.

**Methods:**

A Scopus database search was conducted to identify all Australian gerontology articles published by podiatric authors in English from 1970 to 2024. Bibliometric analysis was performed using an open‐source tool based on the R language. Citations, journals, authors, institutions, and countries were described. Publications were manually categorised according to research type, level of evidence and funding source.

**Results:**

The search strategy yielded 81 eligible articles, which received a total of 5024 citations and were published by 174 authors in 39 journals. The most frequent journal was *Gait and Posture* (12 articles; 15%), and the most published institution was La Trobe University (affiliation of 102 authors). Most of the Australian gerontology articles published by podiatrists focussed on aetiology (*n* = 48; 59%) and only six articles (7%) provided level I evidence. Thirty articles (37%) reported no research funding.

**Conclusion:**

Gerontology remains an underrepresented focus within Australian podiatry research. Despite attracting relatively high citation rates, this field suffers from chronic underfunding and limited research capacity. Investing in dedicated funding and expanding the gerontology research workforce within podiatry is essential to drive innovation, address the growing needs of an ageing population, and strengthen the evidence base for clinical care.

## 1 Introduction

Foot disorders are a major cause of pain, disability, and reduced quality of life in older adults. In Australia, at least one in four people over 65 years of age experience foot pain, most commonly in the forefoot and toes [1]. With the proportion of Australians aged over 65 years projected to reach 24% by 2051, this equates to more than 1.7 million older adults living with debilitating foot problems [2]. Foot pain and deformity significantly impair mobility [3], limit participation in daily activities [4], and increase the risk of balance problems [5] and falls [6]. The Canadian Study of Health and Ageing identified foot problems as having the highest population attributable risk of functional disability in daily living tasks—exceeding the impact of cognitive decline, cardiovascular disease, vision loss, and respiratory disorders [7]. Beyond physical disability, foot pain takes a heavy toll on mental health. Older people with foot pain are more likely to experience anxiety and depression [8] and exhibit lower scores on mental health outcome measures [9].

Podiatry has an important role to play in the assessment and management of foot disorders in older people [10], and there is evidence that podiatric interventions can reduce foot pain [11], improve mobility [12] and reduce the incidence of falls in this population [13]. Indeed, the management of foot problems is integral component of podiatry treatment [14, 15] and the highest proportion of people referred to podiatrists by general practitioners are aged over 65 years [16]. Despite this, management of foot pain in older people is a largely undervalued aspect of health care; and few podiatry students want to specialise in gerontology upon graduation [17]. This reluctance is likely to extend to research, where gerontology attracts only a small pool of investigators and ageing studies are often perceived as less prestigious than other scientific fields [18].

The evidence base of gerontology research in Australian podiatry is unknown. Therefore, as part of a national research priorities initiative [19], this study set out to map the landscape of Australian gerontology research through a comprehensive bibliographic analysis. Our objective was to identify who is driving this research, the nature and location of the work being undertaken, and the sources of funding supporting podiatric gerontology in Australia.

## 2 Method

We conducted a bibliometric analysis of articles between January 1970 and December 2024 using data from the Scopus database (Elsevier, Amsterdam, the Netherlands). The Scopus database was chosen due to its wider coverage of journals compared to PubMed and the Web of Science [20, 21]. The study is reported using the bibliometric analysis guidelines suggested by Montazeri et al. [22].

### 2.1 Search Strategy

The broad search strategy included three concepts: (i) older people, (ii) balance, gait and physical function, and (iii) feet and footwear. Details are provided in Supporting Information S1.

### 2.2 Article Selection

The titles and abstracts of all articles were downloaded from the Scopus database and exported into a systematic review application (Covidence, Veritas Health Innovation, Melbourne, Australia). Titles, abstracts, and full text articles were then independently screened by two researchers (HBM and ANA) with disagreements resolved by discussion or a third researcher (MRC). Eligible publications were original articles or systematic reviews published in English, completed at an Australian education or healthcare institution, in an Australian cohort of participants, where at least one author had an Australian affiliation. For systematic reviews, the first or last author was required to have had an Australian affiliation. Eligible research was deemed to be clinically relevant if it demonstrated applicability to podiatry practice, specifically, if its findings could inform clinical decision‐making or patient care, workforce planning, and continuing education. Laboratory‐based studies or pre‐clinical studies were not included as they were not deemed to be directly relevant to podiatry clinical practice. Guidelines, consensus documents, case studies, research letters, editorials, commentaries and conference abstracts were not included.

### 2.3 Data Extraction

Articles were imported by MRC into Biblioshiny (*bibliometrix* package version 2.2.1; University of Naples Federico II, Naples, Italy) [23]. The following characteristics were extracted from each article: year of publication, journal name, 2023 Impact Factor (using Journal Citation Reports [Clarivate Analytics, Philadelphia, Pennsylvania, USA]), number of citations (as recorded in the Scopus database [Elsevier, Amsterdam, Netherlands]), author names and institutional and country affiliation.

### 2.4 Data Synthesis

Research type was categorised by HBM and ANA according to the United Kingdom Clinical Research Collaboration (UKCRC) Health Research Classification System [24]. This system classifies types of research activities using 48 codes within eight groups: (i) underpinning research, (ii) aetiology, (iii) prevention of diseases and conditions and promotion of well‐being, (iv) detection, screening and diagnosis, (v) development of treatments and therapeutic interventions, (vi) evaluation of treatments and therapeutic interventions, (vii) management of diseases and conditions and (viii) health and social services research. Level of evidence was manually assigned to each study using the National Health and Medical Research Council (NHMRC) criteria [25], which specifies the following: (i) level I: evidence from a systematic review of all relevant randomised controlled trials; (ii) level II: evidence from at least one properly designed randomised controlled trial; (iii) level III: evidence from other well‐designed experimental or analytical studies; (iv) level IV: evidence from descriptive studies. Funding sources were documented according to the Australian Government Higher Education Research Data Collection (HERDC) specifications [26] as follows: (i) category 1: Australian competitive grant research and development income; (ii) category 2: other public sector research and development income; (iii) category 3: industry and other research and development income; (iv) category 4: cooperative research centre research and development income.

## 3 Results

### 3.1 Article Characteristics

**FIGURE 1:**
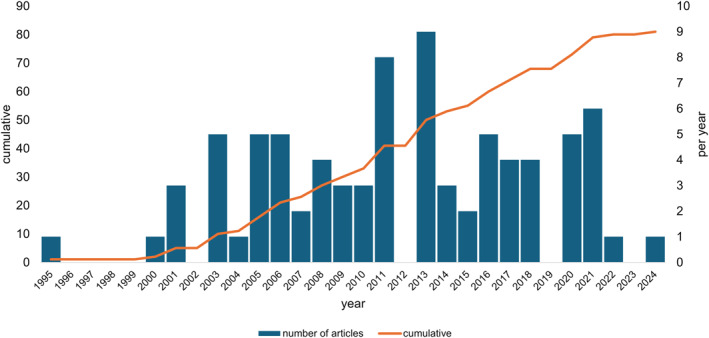
Number of articles (cumulative and per year).

The search strategy yielded 81 eligible articles (see Supporting Information S2). Table 1 presents the characteristics of these articles. The articles received a total of 5024 citations and were published by 177 authors. Table 2 shows that the top 10 most frequent journals were *Gait and Posture*, *Journal of the American Podiatric Medical Association*, *Gerontology, Journal of Foot and Ankle Research, Archives of Physical Medicine and Rehabilitation, BMC Geriatrics, Clinical Biomechanics, Health and Quality of Life Outcomes, Journal of Orthopaedic Research* and *Journal of the American Geriatrics Society*. Figure 1 shows the number of gerontology articles (cumulative and per year) from 1995 to 2024. The highest output occurred in 2011 and 2013.

**TABLE 1:** Publication characteristics.

Years	1995–2023
Total number of articles	81
Original articles	76
Systematic reviews	5
Mean years from publication	12.5
Mean citations per year per article	62.8
Citations	5023
Total authors	174
Mean co‐authors per article	2
International co‐authorships (%)	16
Single‐authored publications	2

**TABLE 2:** Top 10 most frequent journals.

Journal		*n* (%)	Impact factor 2024
1.	*Gait and Posture*	12 (15)	2.4
2.	*Journal of the American Podiatric Medical Association*	7 (9)	0.6
3.	*Gerontology*	5 (6)	3.0
4.	*Journal of Foot and Ankle Research*	4 (5)	2.2
5.	*Archives of Physical Medicine and Rehabilitation*	3 (4)	3.7
6.	*BMC Geriatrics*	3 (4)	3.8
7.	*Clinical Biomechanics*	3 (4)	1.4
8.	*Health and Quality of Life Outcomes*	3 (4)	3.4
9.	*Journal of Orthopaedic Research*	3 (4)	2.3
10.	*Journal of the American Geriatrics Society*	3 (4)	4.5

### 3.2 Authors, Institutions and Countries

Table 3 displays the top 10 most frequent authors of the articles, with the five most published authors being Menz HB, Lord SR, Landorf KB, Spink MJ and Steele JR. Table 4 displays the top 10 most frequent institutional affiliations, with the five most frequently represented institutions being La Trobe University, the University of New South Wales, the University of Wollongong, the University of Sydney and the University of Newcastle.

**TABLE 3:** Top 10 most frequent authors.

Author		*n* (%)
1.	Menz HB	50 (63)
2.	Lord SR	23 (29)
3.	Landorf KB	11 (14)
4.	Spink MJ	10 (13)
5.	Steele JR	10 (13)
6.	Munteanu SE	9 (11)
7.	Munro BJ	8 (10)
8.	Mickle KJ	7 (9)
9.	Morris ME	5 (6)
10.	Zammit GV	5 (6)

**TABLE 4:** Top 10 most frequent institutional affiliations.

Institution		*n*
1.	La Trobe University	102
2.	University of New South Wales	22
3.	University of Wollongong	22
4.	University of Sydney	21
5	University of Newcastle	19
6.	Monash University	13
7.	Prince of Wales Medical Research Institute	9
8.	University of Tasmania	9
9.	University of Melbourne	8
10.	University of Queensland	3

### 3.3 Citations

Table 5 presents the top 10 most highly cited articles according to total citations and citations per year. Articles with the highest total citations were a prospective study of foot and ankle risk factors for falls [27], a cross‐sectional study of foot and ankle characteristics associated with balance [28], a comparison of foot and ankle characteristics in young and older people [29], a cross‐sectional study of footwear characteristics and foot problems in older people [30] and a cross‐sectional study of the contribution of foot problems to mobility impairment [31].

**TABLE 5:** Top 10 cited articles, expressed as total citations and citations/year.

Article		Total citations	Article		Citations/year
1.	Menz HB, Morris ME, Lord SR. Foot and ankle risk factors for falls in older people: a prospective study. The Journals of Gerontology: Series A. 2006 Aug;61 (8):866‐70.	327	1.	McKay MJ, Baldwin JN, Ferreira P, Simic M, Vanicek N, Burns J; 1000 Norms Project Consortium. Normative reference values for strength and flexibility of 1000 children and adults. Neurology. 2017 Jan 3;88 (1):36–43.	18.7
2.	Menz HB, Morris ME, Lord SR. Foot and ankle characteristics associated with impaired balance and functional ability in older people. The Journals of Gerontology: Series A. 2005 Dec;60 (12):1546–52.	325	2.	Menz HB, Morris ME, Lord SR. Foot and ankle risk factors for falls in older people: a prospective study. The Journals of Gerontology: Series A. 2006 Aug;61 (8):866–70.	18.2
3.	Scott G, Menz HB, Newcombe L. Age‐related differences in foot structure and function. Gait & Posture. 2007 Jun;26 (1):68–75.	246	3.	Menz HB, Morris ME, Lord SR. Foot and ankle characteristics associated with impaired balance and functional ability in older people. The Journals of Gerontology: Series A. 2005 Dec;60 (12):1546–52.	17.1
4.	Menz HB, Morris ME. Footwear characteristics and foot problems in older people. Gerontology. 2005 Sep‐Oct;51 (5):346–51.	236	4.	Scott G, Menz HB, Newcombe L. Age‐related differences in foot structure and function. Gait & Posture. 2007 Jun;26 (1):68–75.	14.5
5.	Menz HB, Lord SR. The contribution of foot problems to mobility impairment and falls in community‐dwelling older people. Journal of the American Geriatrics Society 2001 Dec;49 (12):1651–6.	196	5.	Mickle KJ, Munro BJ, Lord SR, Menz HB, Steele JR. ISB Clinical Biomechanics Award 2009: toe weakness and deformity increase the risk of falls in older people. Clinical Biomechanics (Bristol). 2009 Dec;24 (10):787–91.	13.0
6.	Mickle KJ, Munro BJ, Lord SR, Menz HB, Steele JR. ISB Clinical Biomechanics Award 2009: toe weakness and deformity increase the risk of falls in older people. Clinical Biomechanics (Bristol). 2009 Dec;24 (10):787–91.	195	6.	Menz HB, Morris ME. Footwear characteristics and foot problems in older people. Gerontology. 2005 Sep‐Oct;51 (5):346–51.	12.4
7.	Menz HB, Lord SR. Foot pain impairs balance and functional ability in community‐dwelling older people. Journal of the American Podiatric Medical Association. 2001 May;91 (5):222–9.	164	7.	McKay MJ, Baldwin JN, Ferreira P, Simic M, Vanicek N, Wojciechowski E, Mudge A, Burns J; 1000 Norms Project Consortium. Spatiotemporal and plantar pressure patterns of 1000 healthy individuals aged 3–101 years. Gait & Posture. 2017 Oct;58:78–87.	12.0
8.	Menz HB, Munteanu SE. Validity of 3 clinical techniques for the measurement of static foot posture in older people. Journal of Orthopaedic & Sports Physical Therapy. 2005 Aug;35 (8):479–86.	152	8.	Raymond MJ, Bramley‐Tzerefos RE, Jeffs KJ, Winter A, Holland AE. Systematic review of high‐intensity progressive resistance strength training of the lower limb compared with other intensities of strength training in older adults. Archives of Physical Medicine and Rehabilitation. 2013 Aug;94 (8):1458–72.	11.3
9.	Menz HB, Morris ME. Clinical determinants of plantar forces and pressures during walking in older people. Gait & Posture. 2006 Oct;24 (2):229–36.	150	9.	Spink MJ, Fotoohabadi MR, Wee E, Hill KD, Lord SR, Menz HB. Foot and ankle strength, range of motion, posture, and deformity are associated with balance and functional ability in older adults. Archives of Physical Medicine and Rehabilitation. 2011 Jan;92 (1):68–75.	11.1
10.	Spink MJ, Fotoohabadi MR, Wee E, Hill KD, Lord SR, Menz HB. Foot and ankle strength, range of motion, posture, and deformity are associated with balance and functional ability in older adults. Archives of Physical Medicine and Rehabilitation. 2011 Jan;92 (1):68–75.	144	10.	Menz HB, Auhl M, Spink MJ. Foot problems as a risk factor for falls in community‐dwelling older people: A systematic review and meta‐analysis. Maturitas. 2018 Dec;118:7–14.	10.2

### 3.4 Research Types and Level of Evidence

Using the UKCRC criteria, 46 articles (57%) were focused on detection, screening and diagnosis, 22 (27%) on prevention of disease and conditions, 6 (7%) on aetiology and 6 (7%) on underpinning research. There were no studies focussed on development of treatments and therapeutic interventions, evaluation of treatments and therapeutic interventions, management of diseases and conditions or health and social care services research. According to the NHMRC levels of evidence, 6 articles (7%) provided level I evidence, 6 (7%) provided level II evidence, 22 (27%) provided level III evidence, and 47 (58%) provided level IV evidence.

### 3.5 Sources of Funding

Thirty articles (42%) reported no research funding. Of the remaining articles, the most common source of funding reported was from category 1 (*n* = 32; 40%) whereas the least commonly reported was from category 3 (*n* = 9; 11%).

## 4 Discussion

The objective of this study was to undertake a comprehensive bibliometric analysis of Australian gerontology research, describing who conducts it, the types of studies performed, where they are undertaken, and how they are funded. Across 81 eligible articles published between 1995 and 2024, we identified the most active researchers and institutions. Our analysis revealed that gerontology represents only a small fraction of Australian podiatry research, is driven by a limited group of researchers, focuses predominantly on detection, screening, and diagnosis and attracts minimal funding. These findings highlight both the narrow scope and under‐resourced nature of gerontology research in Australia, providing critical insights to guide future investment and workforce development in this field.

The total yield of gerontology articles is the second lowest of the disciplines represented in our larger bibliographic analysis (only surpassing First Nations research) and represents only 5% of research activity relevant to podiatry in Australia [19]. This is somewhat concerning given that older people comprise the vast majority of patients who seek podiatry care [15], and that podiatry plays an essential role in maintaining mobility in this population [10]. Despite this, gerontology research is well‐cited (62.8 citations per year per article)—the highest of any discipline included in our broader analysis [19]. This suggests that although it attracts few researchers (and therefore generates few publications), gerontology research is well‐regarded outside of the profession and addresses fundamental questions of clinical relevance.

Gerontology research was disseminated across a diverse array of journals, many outside the traditional foot and ankle literature. Consequently, clinicians who limit their professional reading to podiatry journals may overlook important research findings. Notably, high‐impact publications often appeared in biomechanics, geriatrics and orthopaedics journals, which typically attract more citations and therefore offer greater visibility for researchers [32]. This misalignment between the dissemination strategies of researchers and the information needs of clinicians highlights a critical gap: ensuring that research reaches its intended audience without compromising the academic impact and career progression of the researchers producing it [33].

Funding of gerontology research relevant to podiatry was extremely limited, with 30 articles (42%) reporting no research funding. This finding is consistent with previous bibliometric analyses [34, 35], and is likely to contribute to the relatively low level of evidence generated by this work. Indeed, according to the NHMRC levels of evidence, only 6 articles (7%) provided level I evidence (systematic review of all relevant randomised controlled trials) and only 6 (7%) provided level II evidence (evidence from at least one properly designed randomised controlled trial). Randomised trials are the cornerstone of high‐quality clinical evidence; yet they are costly and demand substantial funding. Without adequate financial support, generating the robust evidence needed to guide and improve clinical practise remains an ongoing challenge.

These findings should be interpreted with several limitations in mind. First, our analysis excluded guidelines, consensus statements, case studies, research letters, editorials, commentaries and conference abstracts, and therefore, does not capture the full breadth of gerontology‐related output. Second, assigning articles to a single research category is inherently challenging, and some degree of misclassification is possible. Third, reporting of funding is often inconsistent and does not reliably distinguish between project support and salary funding; meaning the true financial investment in this research area may not be accurate. Finally, citation metrics, although widely used, do not necessarily reflect the societal relevance or real‐world impact of research.

Future work should include expanding our bibliometric analysis to other countries to determine whether there are jurisdictional differences in publication activity and funding support, and to repeat this analysis to examine changes over time. Although challenging, it would also be useful to correlate publication activity with clinical outcomes to determine whether research in this area leads to demonstrable improvements for patients.

In summary, our bibliometric analysis highlights that gerontology remains a neglected area within Australian podiatry research. Although studies in this field achieve comparatively high citation rates, they are constrained by limited funding and a small research workforce. Strategic investment in dedicated funding streams and expansion of the gerontology research capacity in podiatry are crucial to foster innovation, meet the rising demands of an ageing population, and enhance the evidence base for clinical practise. These findings provide a foundation for national research priority‐setting to strengthen the quality, impact and clinical relevance of Australian podiatry research.

## Author Contributions

**Hylton B. Menz:** data curation, formal analysis, writing – original draft. **Ameer Nor Azhar:** data curation, writing – review and editing. **Shan M. Bergin:** data curation, writing – review and editing. **Peta E. Tehan:** conceptualization, data curation, formal analysis, writing – review and editing. **Matthew R. Carroll:** data curation, formal analysis, writing – review and editing.

## Funding

This study was funded by the Australian Podiatry Education and Research Foundation.

## Ethics Statement

The authors have nothing to report.

## Consent

The authors have nothing to report.

## Conflicts of Interest

The authors declare no conflicts of interest.

## Permission to Reproduce Material From Other Sources

The authors have nothing to report.

## Supporting information


Supporting Information S1

Supporting Information S2

## Data Availability

The data that support the findings of this study are available from the corresponding author upon reasonable request.
