# Supplementary material for: Australian Podiatry Research in Gerontology: A Bibliometric Analysis
Source: J Foot Ankle Res. 2026 Apr 29;19(2):e70158. doi: 10.1002/jfa2.70158 (PMC13128526; doi:10.1002/jfa2.70158)
Supplement: Supplementary file 1 — Supporting Information S1 [file JFA2-19-e70158-s001.docx]

| 1. Geri* 2. Gero* 3. ‘Older people’ 4. ‘Older adult’ 5. assess* 6. manage* 7. screen* 8. treat* 9. Fall* 10. balanc* 11. Gait 12. function 13. Pain 14. deformit* 15. derm* 16. ‘Foot orth*’ 17. ‘Footwear’ 18. Foot 19. Feet 20. Ankle 21. Knee 22. Hip 23. pod* | |
| --- | --- |
| **Search strategy**  1: 1 OR 2 OR 3 OR 4  2: 6 OR 7 OR 8 9 OR 10 OR 11 OR 12 OR 13 OR 14  3: 15 OR 16 OR 17 OR 18 OR 19 OR 20 OR 21  4: 1 AND 2 AND 3 | |
| **Search restrictions** | |
| *Year* | 1970-2023 |
| *Language* | English |
| *Source* | Article |
| *Author Affiliation* | Australian |
